# A comparative study of the actions of alkylpyridinium salts from a marine sponge and related synthetic compounds in rat cultured hippocampal neurones

**DOI:** 10.1186/1471-2210-7-1

**Published:** 2007-02-02

**Authors:** David J Koss, Kathleen P Hindley, Kanola C David, Ines Mancini, Graziano Guella, Kristina Sepčić, Tom Turk, Katja Rebolj, Gernot Riedel, Bettina Platt, Roderick H Scott

**Affiliations:** 1 School of Medical Sciences, College of Life Sciences and Medicine, Institute of Medical Science, The University of Aberdeen, Foresterhill, Aberdeen AB25 2ZD, Scotland, UK; 2 Laboratorio di Chimica Biooganica, Università di Trento, via Sommarive 14, I-38050 Povo Trento, Italy; 3 Department of Biology, Biotechnical Faculty, University of Ljubljana, Večna pot 111, 1111 Ljubljana, Slovenia

## Abstract

**Background:**

Polymeric alkylpyridinium salts (poly-APS), are chemical defences produced by marine sponges including *Reniera sarai*. Poly-APS have previously been shown to effectively deliver macromolecules into cells. The efficiency of this closely follows the ability of poly-APS to form transient pores in membranes, providing strong support for a pore-based delivery mechanism. Recently, water soluble compounds have been synthesised that are structurally related to the natural polymers but bear a different number of pyridinium units. These compounds may share a number of bio-activities with poly-APS. Using electrophysiology, calcium imaging and 1,6-diphenyl-1,3,5-hexatriene imaging, the pore forming properties of poly-APS and four related synthetic oligomers have been tested on primary cultured rat hippocampal neurones.

**Results:**

Acute application of poly-APS (0.5 μg/ml), reduced membrane potential, input resistance and suppressed action potential firing. Poly-APS evoked inward cation currents with linear current-voltage relationships similar to actions of pore formers on other cell types. Poly-APS (0.005–5 μg/ml) also produced Ca^2+ ^transients in ~41% of neurones. The dose-dependence of poly-APS actions were complex, such that at 0.05 μg/ml and 5 μg/ml poly-APS produced varying magnitudes of membrane permeability depending on the order of application. Data from surface plasmon resonance analysis suggested accumulation of poly-APS in membranes and subsequent enhanced poly-APS binding. Even at 10–100 fold higher concentrations, none of the synthetic compounds produced changes in electrophysiological characteristics of the same magnitude as poly-APS. Of the synthetic oligomers tested compounds **1 **(monomeric) and tetrameric **4 **(5–50 μg/ml) induced small transient currents and **3 **(trimeric) and **4 **(tetrameric) produced significant Ca^2+ ^transients in hippocampal neurones.

**Conclusion:**

Poly-APS induced pore formation in hippocampal neurones and such pores were transient, with neurones recovering from exposure to these polymers. Synthetic structurally related oligomers were not potent pore formers when compared to poly-APS and affected a smaller percentage of the hippocampal neurone population. Poly-APS may have potential as agents for macromolecular delivery into CNS neurones however; the smaller synthetic oligomers tested in this study show little potential for such use. This comparative analysis indicated that the level of polymerisation giving rise to the supermolecular structure in the natural compounds, is likely to be responsible for the activity here reported.

## Background

### Poly-APS bioactivities and transfection

**Figure 1:**
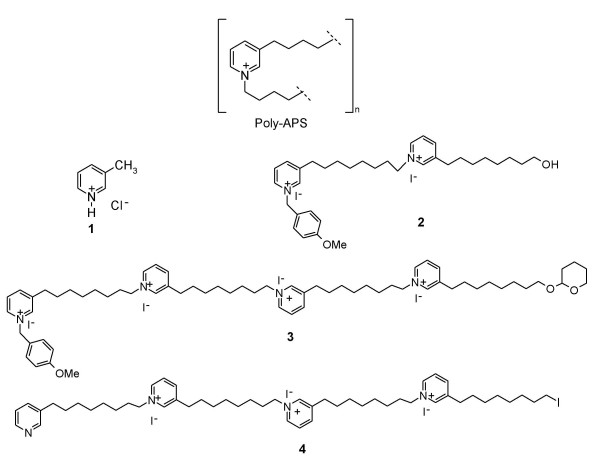
Structures of compounds used in the study. Poly-APS: repeating subunit for the natural poly-APS, characterised as a mixture of polymers with either n = 29 or n = 99. Sample **1**: monomeric synthetic compound. Sample **2**: Dimeric synthetic compound with additional *para*-methoxybenzyl terminal group and a hydroxyl group on the terminal chain. Sample **3**: Tetrameric synthetic compound with additional *para*-methoxybenzyl and tetrahydropyranyl group on the terminal chain. Sample **4**: Tetrameric synthetic compound with iodide as substituent on the terminal chain.

Polymeric 1,3-alkylpyridinium salts (poly-APS), have been isolated from water soluble extracts of the marine sponge *Reniera sarai*. The preparation contains a mixture of oligomeric and polymeric alkylpyridinium compounds but two primary polymeric compounds, one of 5.52 kDa and one of 18.9 kDa are predominant (containing 29 or 99 repeats; Figure 1). Poly-APS are amphipathic molecules expressing both a hydrophilic cationic head group of pyridinium rings and hydrophobic hydrocarbon chains. The existence of dualistic domains within single molecules critically determines their behaviour in aqueous solution and bio-activity [1].

In aqueous solution poly-APS above their critical micelar concentration form irregular spherical aggregates with a mean radius of 23 nm [1,2]. These consist of multiple polymer structures that may form active units of poly-APS. However, most of the observed actions are activities of single polymers since in biological assays the concentration used are well below their critical micelar cencentration. Poly-APS have a number of bio-activities such as anticholinesterase activity [3], haemolysis [4], and cytotoxicity, [5] and many of their actions can be attributed to poly-APS forming pores in membranes.

Electrophysiological recordings from cultured dorsal root ganglia (DRG) neurones indicate that poly-APS and related sponge toxins (halitoxins) collapse membrane potentials and input resistances. These effects result from increases in membrane ionic permeability and reduced ability of cells to maintain electrochemical gradients [6,7]. In the case of poly-APS this pore formation is reversible in most DRG neurones when concentrations below 5 μg/ml were used [6]. This is consistent with data from a previous study by Berlinck and colleagues who showed that halitoxin from *Amphimedon viridis *blocked crutacean nerve action potentials but that partial recovery could be obtained in some experiments [8].

Due to the reversible nature of the pores formed by poly-APS at low μg/ml concentrations, it has been possible to exploit these pores experimentally. For example the transfection of HEK 293 cells with cDNA plasmids for enhanced green fluorescent protein and human tumour necrosis factor receptor 2 has been achieved [9]. Additionally, the pore formation and macromolecule delivery is enhanced at 12°C compared to 21°C. These findings indicate that pore formation but not endocytosis is a requirement for efficient macromolecule delivery [10].

It is of great interest that synthetic "poly-APS like" monomers and oligomers have been produced. These compounds express one or more pyridinium rings ranging from monomers to tetramers, and varying hydrocarbon chain length (Figure 1 structures **1–4**). Some bio-activities of these synthetic compounds, including: antibacterial, haemolysis, cholinesterase inhibition and to a lesser extent protein phosphate 2A inhibition, have already been characterised. In particular these compounds show some correlation between the length of the polymer (degree of polymerization) and functional activity [11].

In this study we have aimed to characterise and compare the pore-forming properties of poly-APS and four synthetic compounds using primary cultures of rat hippocampal neurones and evaluate the potential for intracellular macromolecule delivery into CNS neurones by any of the compounds.

## Results

### Acute electrophysiological actions of poly-APS on cultured hippocampal neurones

**Figure 2:**
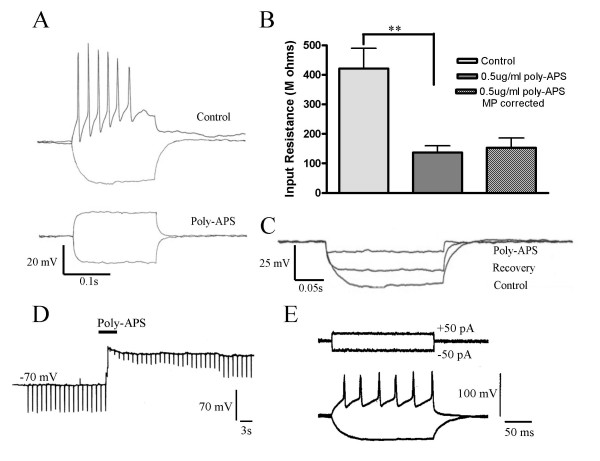
Poly-APS (0.5 μg/ml) reversible elevated ionic permeability and suppressed neuronal excitability in cultured hippocampal neurones. a) Electrotonic potentials in response to +/- 100 pA current step. Multiple action potential firing was seen in response to +100pA. In the same neurone after 5 minutes application of 0.5 μg/ml poly-APS the electrotonic potential to -100 pA was reduced and action potential activation was depressed. B). Application of poly-APS greatly reduced the input resistance. This reduction was still observed even when membrane potential changes were corrected (n = 6 for control and poly-APS and n = 5 for membrane potential (MP) corrected (** = P < 0.01 student paired t-test). C). Voltage records showing the response to poly-APS and partial recovery of the input resistance after 45 minutes. D) Voltage record showing membrane potential and electrotonic potentials (downward deflections) evoked by -80 pA current pulses and the onset of the response to a brief application of poly-APS (0.5 μg/ml). The development of partial recovery can also be seen. E) Voltage records showing action potential firing evoked by a +50 pA step command and an electrotonic potential evoked by a -50 pA step command. These responses were evoked from a hippocampal neurone that had been exposed to 0.5 μg/ml poly-APS and then placed back in culture for 24 hours.

Low-pressure ejection of 0.5 μg/ml poly-APS collapsed membrane potential from a resting level of -65 ± 3 mV to -16 ± mV (n = 6; *p *< 0.01). This effect was accompanied by suppression of action potential firing evoked by +100 pA current step commands and reductions in electrotonic potentials evoked by -100 pA current step commands (Figure 2A). Under control conditions -100pA current step commands evoked a mean hyperpolarising electrotonic potential of 44.3 ± 7.5 mV, which equated to a mean input resistance of 443 ± 75 MΩ. After application of poly-APS for 3 minutes, the mean input resistance was significantly reduced to 137.1 ± 24.2 MΩ (n = 6; *p *< 0.01). Adjusting for the reduction in membrane potential using constant current injection, resulted in an input resistance of 153.6 ± 37.4 MΩ, which was not significantly different from non-adjusted values (n = 5; *p *> 0.05, Figure 2B).

Prolonged intracellular recordings revealed a variable timeline of pore reversibility and cell recovery from poly-APS (0.5 μg/ml). In one example neurone, approximately 45 minutes after application of poly-APS, a partial recovery of membrane potential and input resistance was observed (Figure 2C). Prior to poly-APS application the resting membrane potential was -65 mV and the input resistance was 308 MΩ. After 5 minutes application of poly-APS, resting membrane potential and input resistance fell to -18 mV and 115 MΩ respectively, before recovering to -63 mV and 168 MΩ. However, injection of +100 to +300 pA current step commands failed to elicit action potential firing. Whilst this indicates some reversibility in the membranes ionic permeability, it suggests that suppression of action potentials firing is not solely due to the collapse of input resistance but may involve more prolonged inactivation of voltage-activated channels and loss of electrochemical gradients. Figure 2D shows a record of the onset of the response to brief application of 0.5 μg/ml of poly-APS and the development of recovery after membrane potential and input resistance collapse. Preliminary data (n = 4), indicated that if cultures were exposed to poly-APS (3 minutes, 0.5 μg/ml) and then returned to culture conditions for 24 hours at least some neurones recover and have the ability to fire action potentials when stimulated (Figure 2E).

**Figure 3:**
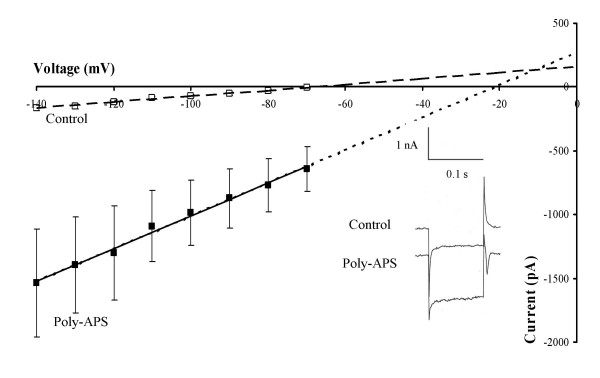
Poly-APS activated inward currents in cultured hippocampal neurones. Mean current- voltage relationships plotted under control conditions (broken line) and after activation of inward current by 0.5 μg/ml poly-APS (solid line). Poly-APS application produced an inward cation current with a linear current/voltage relationship. Inset records show currents evoked by step depolarisations to -140 mV from a holding potential of -70 mV under control conditions and in the presence of poly-APS.

To further characterise the actions of poly-APS, neurones were voltage clamped at potentials between -70 and -140 mV in the absence and presence of the natural product preparation. Figure 3 shows the linear current-voltage relationships generated under control conditions and in the presence of poly-APS. Leak subtractions of linear leak current and capacitance were performed to generate values for the net poly-APS activated-currents. These showed a linear correlation with voltage of R^2 ^= 0.94 ± 0.03, a mean slope conductance of 10.5 ± 3.3 nS. A mean reversal potential of 10 ± 10.5 mV (n = 9) for the poly-APS-activated current was obtained from individual experiments by plotting difference current (after leak subtraction)-voltage relationships and identifying the point at which the linear relationship crosses the x-axis (I = 0).

The poly-APS-evoked currents displayed typical properties of non-specific cation currents resulting from permeation of the cell membrane.

### Electrophysiological response to application of synthetic alkylpyridinium compounds

**Figure 4:**
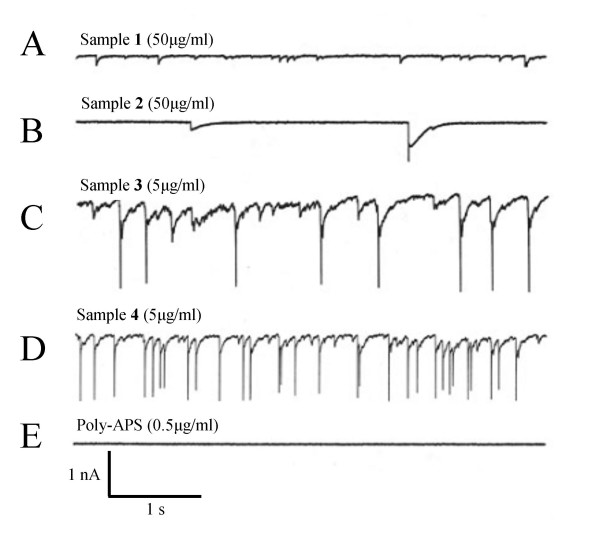
Poly-APS and the related synthetic compounds produced distinctive electrophysiological activity. 5-second traces of current activity recorded during the application of A) sample **1**, B) sample **2**, C) sample **3**, D) sample **4**, and E) poly-APS by low-pressure ejection. A-D, Synthetic compounds **1–4 **produced two distinct forms of activity. Activity was not sustained and stable baselines were reached in all cells. Poly-APS produces no spontaneous activity (action currents).

Each synthetic alkylpyridinium compound was applied to cultured rat hippocampal neurones at one of two concentrations and attempts were made to maintain similar numbers of pyridinium rings in the different solutions applied (Table 1). From a holding potential of -70 mV the monomer and dimer, sample **1 **and **2 **respectively, evoked only small non-uniform transient inward currents. The peak current amplitude of events induced by sample **1 **were < -0.2 nA in size, with duration of < 100 ms. Sample **2 **induced transient inward currents at a lower frequency, but with varying amplitudes ranging from between -0.2 nA and -1 nA, with a greater duration lasting approximately 300 ms (Figure 4A &4B ). These currents were recorded within the first few seconds of application of the test compounds but inward current activity stopped with persistent application of the test compounds.

**Table 1:** Concentrations of the samples: the values in μg/ml were used in this study to give approximately equivalent numbers of pyridinium rings for each compound investigated. The corresponding values in μM are given below

	**Molecular Weight**	**0.005 μg/ml corresponds to (μM)**	**0.05 μg/ml corresponds to (μM)**	**0.5 μg/ml corresponds to (μM)**	**5 μg/ml corresponds to (μM)**	**50 μg/ml corresponds to (μM)**
Poly-APS	12200^a)^	4* 10^-4^	4* 10^-3^	0.04	0.4	4
Monomer S1	129	3.8* 10^-2^	0.38	3.8	38	388
Dimer S2	772	6.5* 10^-3^	0.065	0.65	6.5	65
Tetramer S3	1490	3.4* 10^-3^	0.034	0.34	3.4	34
Tetramer S4	1268	3.9* 10^-3^	0.039	0.39	3.9	39

^a) ^Mean molecular weight considering natural poly-APS as a 1:1 mixture of the two polymers with n = 29 and n = 99.

The larger synthetic compounds, tetramers **3 **and **4**, both produced more sustained inward currents and evoked action currents of -1.5 to -2 nA (Figure 4C &4D). In contrast, the inward currents evoked by samples **1 **and **2 **rarely evoked regenerative action currents and did not produce the high frequency discharge activity. Although poly-APS produced the largest and most sustained inward currents, they suppressed evoked action potential firing and did not support spontaneous action potential discharges (Figure 4E).

The actions of the synthetic compounds on current-voltage relationships (range -70 to -140 mV in 10 mV intervals) were also investigated. Samples **1, 2, 3 **and **4**, were tested at concentrations of 5 and 50 μg/ml.

**Figure 5:**
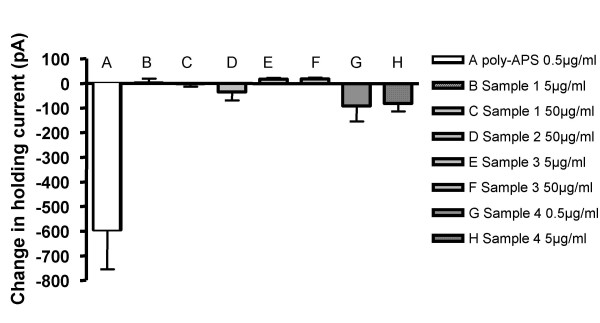
Poly-APS evoked larger and more sustained inward currents than did the synthetic compounds. Bar chart showing changes in holding current, at a holding voltage of -70 mV, induced by application of poly-APS and samples **1–4**. Only poly-APS (open bar A) at 0.5 μg/ml produced a significant inward current (n = 10; P < 0.01 paired t test) compared to resting membrane holding current.

Firstly, we compared the net currents evoked at a holding voltage of -70 mV, the mean data for all samples are shown in Figure 5. Poly-APS (0.5 μg/ml) evoked a highly significant increase in holding current while application of sample **1 **at 5 μg/ml did not significantly alter the holding current. Under control conditions the mean holding current was -44 ± 8 pA compared to -42 ± 10 pA (n = 3; *p *> 0.05) in the presence of sample **1 **(5 μg/ml). At 50 μg/ml sample **1 **also did not alter the mean holding current at -70 mV (-65 ± 26 pA compared to -67 ± 19 pA (n = 6; *p *> 0.05). Sample **2 **at 50 μg/ml also did not significantly alter holding current (n = 4; *p *> 0.05). Sample **4, **the tetramer compound and structurally most like poly-APS, also did not change the holding current when tested at 0.5 and 5 μg/ml (n = 4 & 5; *p *> 0.05). However, sample **3 **produced a significant decrease in holding current when applied at 5 μg/ml. The holding current decreased from a mean control value of -70 ± 21 pA to -53 ± 17 pA (n = 4; *p *< 0.05). Similarly, at 50 μg/ml sample **3 **also decreased holding current from -99 ± 34pA to -82 ± 39 pA (n = 5; *p *< 0.05).

Secondly, we considered the actions of the samples on current-voltage relationships between -70 and -140 mV. Comparative responses produced by the different samples were statistically analysed using a 2-way ANOVA test and comparing individual sample responses against controls at corresponding voltages. Application of poly-APS at 0.5 μg/ml produced a significant effect on the current-voltage relationship (n = 4; *p *= 0.0001), which is described in Figure 3. The Bonferroni post test revealed significant increases in holding current at all clamped potentials (n = 4; *p *< 0.01 for -70 and -80 mV, *p *< 0.001 for -90 to -140 mV).

When applied at 5 μg/ml, sample **1 **produced significant effects on the current-voltage relationship that were evident at -140 mV. The post-test confirmed a modest but significant increase in holding current at -140 mV (-191 ± 85 pA under control conditions compared with -274 ± 54 pA in the presence of 5 μg/ml sample **1 **(n = 3; *p *< 0.05). Similarly, sample **4 **tested at 0.5 and 5 μg/ml also produced a modest but significant increase in the slope of the current-voltage relationship (n = 4, 5; *p *< 0.01). However, sample **2 **at 50 μg/ml produced no change in the current-voltage relationship (n = 4). In contrast, sample **3 **tested at 50 μg/ml showed a modest but significant decrease in the slope of the current-voltage relationship between -90 and -140 mV (n = 5; *p *< 0.01). The synthetic compounds tested showed modest or no significant sustained effects on the linear current-voltage relationship under voltage clamp. In summary, despite subtle transient changes in holding current, none of the synthetic samples produced currents of the magnitude and duration as those evoked by poly-APS (n = 3–5; *p *< 0.01), even when the synthetic samples were applied at 100 times the concentration of poly-APS.

### Intracellular Ca^2+ ^imaging analysis of the Ca^2+ ^transients evoked by poly-APS and the synthetic compounds

**Figure 6:**
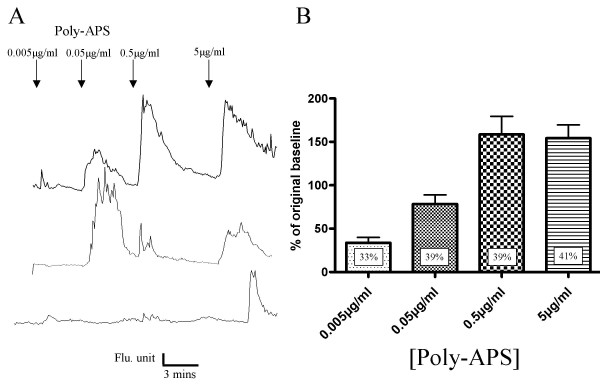
Poly-APS evoked increases in intracellular Ca^2+^. A) Example traces from individual neurones exposed to different concentrations of poly-APS during the same Ca^2+ ^imaging experiment. Three different levels of response to poly-APS are seen. The top trace shows the most typical responses. B) Bar chart showing relative increases in intracellular Ca^2+ ^in response to increasing concentrations of poly-APS sampled from a total of 106 neurones. Data shows trend for dose-response relationship (** = P < 0.01 ** = P < 0.001). Percentage values are the % of neurones responding to each concentration of poly-APS.

**Figure 7:**
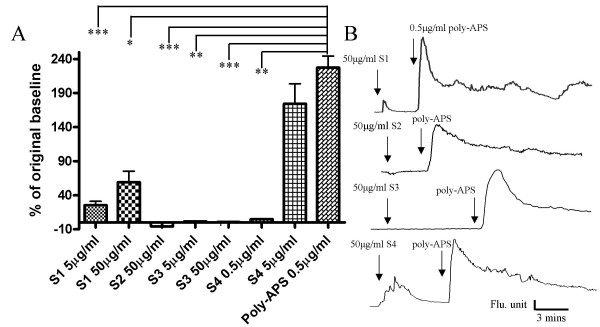
Synthetic compounds **1–4 **produced smaller changes in intracellular Ca^2+ ^compared to poly-APS. A) Bar chart showing mean percentage changes in fluorescence compared to baseline values. Samples **1**, **2 **and **3 **produced modest changes in intracellular Ca^2+ ^that were significantly smaller than those seen with poly-APS (*P < 0.05, **P < 0.01 & ***P < 0.001). Sample **4 **at a concentration of 5 μg/ml gave a similar response to 0.5 μg/ml poly-APS. B) Example traces of mean responses to the synthetic compounds and poly-APS. Each trace is comprised of the mean from 17–34 neurones.

Poly-APS were applied in sequential ascending concentrations of 0.005, 0.05, 0.5 and 5 μg/ml. The responses recorded from hippocampal neurones showed that poly-APS transiently increased intracellular Ca^2+^. However, poly-APS produced a variety of distinct concentration response relationships. Three distinct poly-APS response profiles can be seen in Figure 6A. In most neurons observed (~77% of responding neurons) the amplitudes of the intracellular Ca^2+ ^transients increased with increasing poly-APS concentrations. However in a second group with low sensitivity, responses were only seen to the highest concentration (5 μg/ml) of poly-APS. A third group of neurones responded to 0.5 and 5 μg/ml poly-APS but not to the lowest or intermediate concentrations. When data from all cells were pooled the poly-APS induced Ca^2+ ^rise displayed a concentration-dependent response relationship. At concentrations of 0.005, 0.05, 0.5 and 5 μg/ml poly-APS produced 33 ± 6, 78 ± 5, 159 ± 20 and 154 ± 15 % increase in Ca^2+ ^relative to baseline levels (n = 10; *p *< 0.001 Kruskal-Wallis). Significant increases in intracellular Ca^2+ ^levels were observed when comparing responses to 0.005 μg/ml poly-APS with responses to 0.5 and 5 μg/ml (n = 10; *p *< 0.001), and 0.05 μg/ml with 0.5 and 5 μg/ml (n = 10; *p *< 0.01; Figure 6B). Analysis of responders revealed a poly-APS sensitive subpopulation of cultured hippocampal neurones, with 41 % responding to 5 μg/ml poly-APS and 30 – 40 % responding to the lower concentrations tested. This reflects a consistent threshold sensitivity to low concentrations of poly-APS in a subpopulation of hippocampal neurones. In most cases the same cells are responding to different concentrations of poly-APS. However, some cells that respond to low concentrations of poly-APS failed to recover preventing further analysis. The variation in response may be a result of a heterogenous population of neurons within the hippocampal cultures, whilst the majority of neurones are likely to be pyramidal cells it is also likely that various interneurones exist within the culture. Differences in membrane lipid composition may alter the sensitivity of neurones to poly-APS. The synthetic compounds were also applied to hippocampal cultures and changes in intracellular Ca^2+ ^measured. Similar to the electrophysiology results with the synthetic compounds, direct comparisons with poly-APS (0.5 μg/ml) were made based on using concentrations that contained equivalent numbers of pyridinium rings. Poly-APS (0.5 μg/ml) were also applied after synthetic compounds to test for the sensitivity of the neurones. Interestingly, only those neurones which responded to poly-APS, produced a Ca^2+ ^transient in response to application of the synthetic compounds. Of these compounds, only samples **1 **and **4 **produced significant increases in intracellular Ca^2+ ^but samples **1, 2 **and **3 **at 50 μg/ml all gave significantly smaller responses compared to 0.5 μg/ml poly-APS (n>3; *p *= 0.01 to 0.001). Only 14 % and 37 % of neurones responded to 5 μg/ml and 50 μg/ml sample **1**. Sample **1 **(5 and 50 μg/ml) produced increases in Ca^2+ ^of 25.3 % and 59 % compared to baseline levels. The increases in intracellular Ca^2+ ^evoked by sample **1 **(5 & 50 μg/ml) were significantly smaller (11 and 26 %; n = 12 & 14) compared to the mean response evoked by 0.5 μg/ml poly-APS (n = 96; *p *< 0.001). All the neurones that responded to 0.5 μg/ml poly-APS also responded to 5 μg/ml of sample **4**. Sample **4 **at 5 μg/ml produced a 174 % rise in Ca^2+ ^relative to the baseline levels and gave a mean response of 77 % of that induced by 0.5μg/ml poly-APS (n>3; *p *< 0.001). Figure 7 summarises the Ca^2+ ^imaging data and shows maximal responses to the synthetic samples compared to increases in intracellular Ca^2+ ^evoked by poly-APS.

### DPH Fluorescence imaging

**Figure 8:**
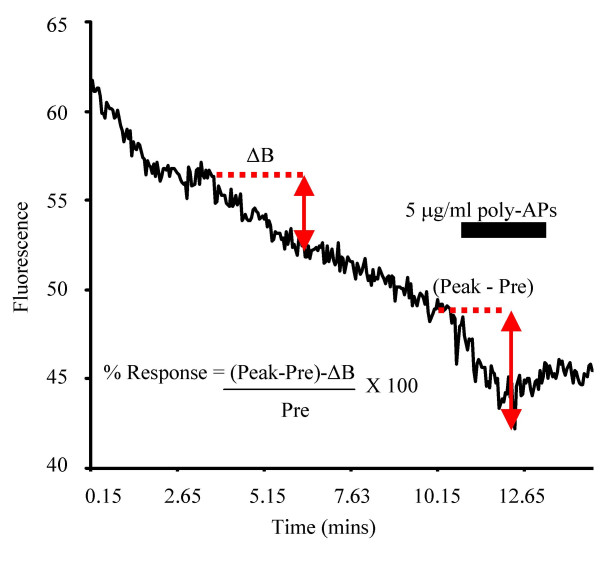
DPH membrane fluorescent measurements. An example record of a poly-APS response showing the measurements made (red text). Changes in membrane fluorescence were quantified relative to pre-drug application fluorescence. The change of fluorescence intensity was measured for each region of interest as the peak response minus pre-drug fluorescence (Peak-Pre). For each individual region of interest the pre-drug baseline fluorescence decay was measured from two time points prior to the application of any drug, this provided a base change of DPH intensity (ΔB). Subtraction of ΔB from peak pre-drug response (see equation) allowed all responses to be normalised for the decay rate of DPH. Data was finally calculated as a % change of pre-drug baseline, see inset equation. All measurements were made from individual raw records that were analysed without filtering or smoothing.

Membrane fluidity was quantified using the lipophilic fluorescence probe 1,6-diphenyl-1, 3, 5-hexatriene (DPH). Initial levels of DPH fluorescence within the membrane are dependent upon the ratio of lipids, cholesterol and proteins within the bilayer. Insertions of molecules into the membrane that are lipophilic increase the fluorescent signal and indicate a more fluid membrane environment. However, insertion of non-lipophilic molecules that can form lipophobic or hydrophilic regions in the membrane may make the membrane more rigid and decrease the lipid density [12-14]. This results in a reduced DPH fluorescence indicative of a reduction in membrane fluidity (Figure 8).

**Figure 9:**
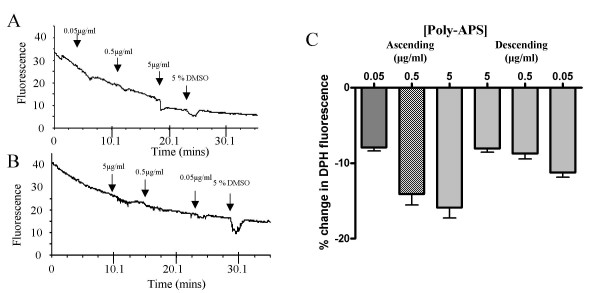
Cumulative dose-response relationships showing the actions of poly-APS on membrane fluidity. A) Mean record of changes in fluorescence (membrane fluidity, n = 15 from a single experiment) in response to increasing concentrations of poly-APS (2 mins application per concentration). The change in density is partially reversible on washing. B) Mean record of changes in fluorescence in response to decreasing concentrations of poly-APS (starting with the highest 5 to 0.05 μg/ml, n = 8 from a single experiment). C) Bar chart showing the mean responses to poly-APS (n = 103) when the order of application of different concentrations of poly-APS was varied. Regardless of the order of application, there is a trend for an increase in % change in membrane fluidity.

Application of poly-APS in order of ascending concentration, 0.005 to 5 μg/ml, produced transient reductions in DPH fluorescence, the magnitudes of which increased with higher concentrations (Figure 9A). When applied at 0.05, 0.5 and 5 μg/ml, poly-APS decreased fluorescence by 8, 14 and 16 % respectively. The response to 0.05 μg/ml poly-APS was significantly lower than that produced by 5 μg/ml poly-APS (n = 3; *p *< 0.001). The responses to poly-APS appeared to display a straightforward dose-dependent relationship. However, when applied in order of descending concentrations (5, 0.5 & 0.05 μg/ml), poly-APS produced cumulative responses (Figure 9B). Both 0.05 and 5 μg/ml poly-APS produced significantly different decreases in fluorescence, depending on the sequences of application. Poly-APS (0.05 μg/ml) decreased fluorescence by 9 % and 11 % when applied in ascending and descending order (n = 3; *p *< 0.001). In contrast, 5 μg/ml poly-APS decreased fluorescence by 16 % compared to 11 % when applied in ascending and descending orders (n = 3; *p *< 0.001; Figure 9C).

### Surface plasmon resonance data for poly-APS

Surface Plasmon Resonance (SPR) is a method that uses changes in light reflection off a metal surface coated with a target ligand to detect binding and dissociation of a test analyte. When analyte is associated with the ligand, it alters the refractive index which can be measured and used to determine the time course of association and dissociation. In this study we have used SPR to detect interactions between surface-immobilized cholesterol/sphingomyelin lipid vesicles and poly-APS. Given our previous results and anomalous dose-response relationships we were particularly interested in determining whether Poly-APS have a reversible association with lipids.

**Figure 10:**
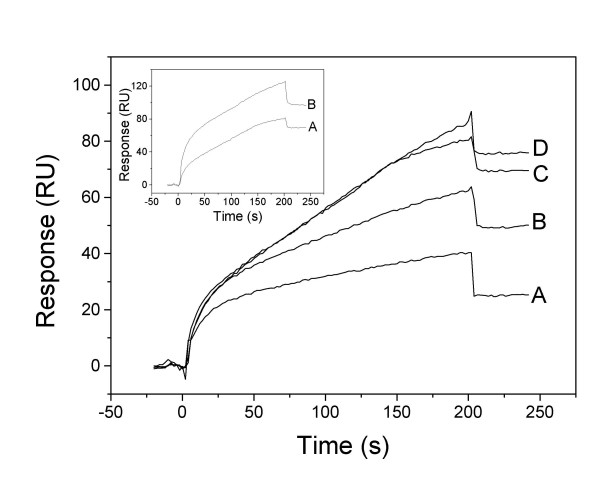
Representative sensorgrams showing poly-APS binding to sphingomyelin/cholesterol (1/1) LUVs. 100 μl of poly-APS, dissolved in 140 mM NaCl, 20 mM Tris.HCl, 1 mM EDTA, pH 8.0, were injected at 30 μl/min for 3.3 min over the LUVs coupled to a Sensor Chip L1 at 25°C. Poly-APS were applied in the following order: 0.005 (A), 0.005 (B), 0.5 (C), and 0.5 (D) μg/ml. *Inset*: Sensorgrams showing the binding signal (A) of 0.5 μg/ml poly-APS applied over the freshly deposited sphingomyelin/cholesterol (1/1) LUVs, and (B) the binding signal to the same LUVs after 6 successive injections of poly-APS (0.5 μg/ml). The SPR experiments were run in triplicate.

SPR sensorgrams corrected for bulk effects, displaying the association-dissociation kinetics of poly-APS interaction with cholesterol/sphingomyelin (1/1) large unilamellar vesicles (LUVs) immobilized on L1 chip, are shown in Fig. 10. Increasing kinetics during the association phase (3.3 min) was observed with increasing concentrations suggesting dose-dependent behaviour. However, the binding of poly-APS did not reach the equilibrium during the 3.3 min application period. Approximately 80% of the signal persisted during the dissociation step, indicating that a considerable amount of poly-APS remained irreversibly inserted or attached to the lipid bilayer. Moreover, successive injections of poly-APS produced a accumulative binding effect. For example, the binding signal obtained after successive treatment of the bilayer with 6 doses of 0.5 μg/ml poly-APS was enhanced by 40% in comparison with the first injection over the freshly deposited LUVs (Fig. 10, *inset*). These repeatedly observed effects indicate that poly-APS molecules already present in the membrane can enhance further binding of poly-APS, and support the data obtained with calcium imaging and DPH (Fig. 6 & 9).

## Discussion

The electrophysiological actions of 0.5 μg/ml poly-APS on cultured hippocampal neurones were consistent with pore formation by the sponge toxins and are similar to the effects previously seen in HEK293 cells and DRG neurones [6,9]. Additionally, we again observed that within any of the populations of cultured cells there was a considerable variability in sensitivity to poly-APS. In the cases of DRG and hippocampal neurones this may be explained by the heterogeneous nature of the cultures, but some variability is also observed in HEK293 cells. For example, considering evoked Ca^2+ ^transients in hippocampal neurones, 39 % of neurones responded to 0.5 μg/ml poly-APS but this value only increased to 41 % when a 10 fold higher concentration was used. Although all HEK 293 cells responded to 5 μg/ml poly-APS there was great variability in the threshold sensitivity to this natural compound [9]. Additionally, there is also a large degree of variability in the currents and Ca^2+ ^transients evoked by poly-APS. This may relate to the compositions of plasma membranes providing varying access and/or numbers of binding sites for the polymeric units to interact with. In this study we have also revealed a level of variability in poly-APS responses (both in Ca^2+ ^transients and membrane fluidity measurements) that related to previous exposure to the natural product. One interpretation of the findings is that poly-APS polymers do not fully wash out of membranes but membrane re-modelling takes place to facilitate repair but disrupt pores. A consequence of this would be that poly-APS molecules already within the membranes influence the responses produced by subsequent reapplication of poly-APS. This may account for the complex dose-response relationships seen in this study that depended on the order of application of different concentrations of poly-APS.

Recovery of hippocampal neurones from both electrophysiological responses and in some cases Ca^2+ ^transients evoked by poly-APS was slow and often incomplete. Faster (20 minute time frame) and greater levels of recovery (both in numbers {97 %} of cells and amplitudes) were seen in DRG neurones exposed to 0.5 μg/ml poly-APS [6]. This may be due to variations in Ca^2+ ^homeostasis in different neurones and predisposition to Ca^2+ ^induced excitotoxicity. However, preliminary data suggests that 24 hours after exposure to poly-APS, at least some hippocampal neurones show normal electrophysiological properties and fire action potentials.

### Activity of synthetic compounds

Although polymeric alkylpyridinium compounds may be useful as transfection reagents and for intracellular delivery of macromolecules, there are clear hurdles that restrict progress. To date research concerning these natural metabolites has focused on the isolation from marine sponges of natural products as cocktails and not on single pure compounds. Purification of individual polymeric alkylpyridinium compounds from nature has proved problematic because of availability and variability of materials and the complex nature of the mixtures that may contain tens of molecules with similar molecular weights and related structures [7]. Additionally, poly-APS for example might be improved on because they have recently been found ineffective for the intracellular delivery of siRNA [10]. Ideally, total synthesis of defined pure alkylpyridinium polymers would be a way forward. Although making large polymers by an efficient and controlled synthetic sequence remains a goal, a series of water-soluble monomers and oligomers, the later -as short "poly-APS-like" examples, have been made. Analysis of chemical structure and bioactivity has suggested several factors that may influence the extent of membrane interaction including: 1) distribution of positive charges, 2) alkyl chain length and 3) numbers of pyridinium rings. These properties partially correlate with antibacterial properties. Although sample **4 **showed greater activity than the other synthetic compounds used in this study it also shows greater antibacterial activity than poly-APS [11]. In our study, higher levels of membrane perturbation were seen with sample **4 **and to some extent with sample **3 **compared with relative inactivity seen with samples **1 **and **2**. Action potential discharges and Ca^2+ ^transients evoked by sample **4 **particularly reflected this. Structural variation may account for the distinct sets of transient events evoked by the synthetic compounds and may provide indications of extents of the compounds surface interactions resulting in the alteration of membrane charge separation through screening or capacitance changes. The transient nature of the events induced by sample **1 **and **2 **may indicate incomplete membrane interactions such as partial intercalation rather than pore formation. Whilst the more prolonged disruption of holding current induced particularly by sample **4 **may indicate more stable interactions with membranes. Interaction with specific membrane proteins such as ionic pumps and channels may also contribute to the initial small transient responses observed. In this study, sample **4 **produced the more robust responses compared to sample **3, **yet they share very similar structural features, suggesting that the presence of additional functional groups on sample **3 **inhibits membrane or protein interactions that lead to activity. In contrast to two of the synthetic compounds (**3 **and **4**), poly-APS did not produce action potential discharges and actually suppressed action potential firing. This was most likely due to the larger decrease in input resistance, to a point where regenerative excitability is prevented.

Considerable haemolytic activity was previously seen with poly-APS inducing lysis of bovine erythrocytes with an approximately 100 fold higher effect than compound **4 **[11]. Our data from cultured hippocampal neurones was consistent with the findings previously made by Mancini *et al*. who have shown that haemolytic, antibacterial and anticholinesterase activities of alkylpyridinium compounds mainly increase with higher degrees of oligomerisation, numbers of positive charges and pyridinium rings [11].

### DPH-membrane fluidity

Analysis of the dynamic changes in membrane fluidity in response to poly-APS provided new insights into possible mechanisms of pore formation and cell recovery after pore formation. Poly-APS decreased membrane fluidity but again showed complex dose-response relationships that were greatly influenced by the order in which different concentrations of natural product were applied. The cumulative type dose-response relationship may result from alkylpyridinium compounds remaining within the lipid membranes, even after the structure has recovered from pore formation. This was striking, when viewing the raised levels of membrane disruption produced by 0.005 μg/ml poly-APS, applied after the neurones had been exposed to higher concentrations of pore-former. We speculate that poly-APS within the membrane that are not producing an increase in permeability provide either co-operative support for further interactions between membranes and poly-APS, or produce sites of membrane vulnerability. This increase in membrane vulnerability may relate to partial membrane repair and increased availability of poly-APS binding sites and thus prime the membrane for further poly-APS interactions. The preliminary SPR data in the present study support these ideas and showed that a significant proportion of poly-APS remains in or bound to membranes after washing. Furthermore the repeated application of poly-APS to membranes results in accumulation of associated poly-APS and that accumulation of poly-APS in membranes enhances further binding of poly-APS. The nature of this molecular interaction may have practical implications for the refinement of the transfection protocol utilising poly-APS, where successive applications of low concentration of poly-APS may prove less cytotoxic than a single application of poly-APS at higher concentrations.

## Conclusion

Poly-APS, a naturally occurring mixture of two polymeric alkylpyridinium compounds produces pore formation in cultured hippocampal neurones that can be identified and to some degree quantified using electrophysiology, Ca^2+ ^and DPH imaging. However, related water-soluble synthetic compounds ranging from monomers to tetramers produced either no pore formation or modest changes in membrane permeability. Over the time courses of the experiments conducted in this study it appears that the synthetic oligomers do not produce similar effects to poly-APS. The data indicate that larger synthetic oligomers with a greater degree of polymerisation than the synthetic molecules used in this study will be required to mimic poly-APS and potentially be useful for macromolecule delivery into hippocampal neurones. Intracellular delivery of macromolecules via poly-APS-induced pores may still prove problematic because of the levels of recovery and the requirement for prolonged exposure to pore formers.

## Methods

### Solutions and test compounds

The extracellular NaCl-based medium used in electrophysiological experiments contained in mM; 130 NaCl, 3 KCl, 2 CaCl_2_, 0.6 MgCl_2_, 1 NaHCO_3_, 10 N-(2-hydroxyethyl) piperazine-N-ethanesulfonic acid (HEPES) and 4 glucose; pH was adjusted to 7.4 with addition of NaOH and osmolarity adjusted with sucrose to 310–320 Osm/ml.

The intracellular patch pipette solution contained in mM; 140 KCl, 5 ethylene glycol bis(2-aminoethyl ether)-N, N, N'N'-tetraacetic acid (EGTA), 0.1 CaCl_2_, 2 MgCl_2_, 10 HEPES and 2 adenosine triphosphate (ATP); pH was adjusted to 7.2 with addition of Tris and osmolarity adjusted with sucrose to ~310 Osm/ml.

The HEPES buffered saline solution (HBS) contained in mM; 130 NaCl, 5.4 KCl, 1.8 CaCl_2_, 1 MgCl_2_, 10 HEPES and 25 D-glucose; pH was adjusted to 7.4 with addition of NaOH.

Protease solution contained 3 mg protease X (Sigma), 3 mg protease XIV (Sigma) dissolved in 3 ml HBS.

Minimum essential medium (MEM) contained 27 ml MEM (Invitrogen), 3 ml foetal bovine serum (Gibco) and 300 μl L-glutamine.

Neurobasal medium contained 29 ml neurobasal medium (Invitrogen), 300 μl L-glutamine, 600 μl bovine serum albumin (BSA) and 50 mM glutamate.

The polymeric 3-akylpyridinium salts (poly-APS) were isolated and purified from the marine sponge *Reniera sarai *as previously reported [1]. A 5 mg/ml stock solution in distilled water was stored at -20°C. The synthetic "poly-APS-like" samples **1, 2, 3 **&**4 **were synthesised as previously reported [11]. The structural peculiarities on the pyridinium moieties arise from the synthetic strategy adopted. Experimental concentrations of 0.5 – 50 μg/ml were prepared either in NaCl- based medium for electrophysiology or HBS for Ca^2+ ^imaging.

### Cell culture

Hooded Liester Neonatal rat pups (1–3 days post natal) were killed by means of neck dislocation and hippocampi dissected out and placed in ice-cooled HBS. Hippocampi were placed in protease solution and mechanically dissociated by finely chopping the tissue. The tissue remained in the solution being enzymetically dissociated for 40 minutes.

In a sterile hood, tissue was removed from the protease solution and washed with 1.5 ml HBS and partially dissociated by trituration. The tissue was re-suspended in 1.5 ml HBS and then centrifuged at 1600 rpm for 3 minutes. The supernatant was removed and replaced with 1.5 ml of MEM solution previously warmed to 37°C. The tissue was again triturated and the cell suspension was then spun at 1500 rpm for 2 minutes. The supernatant was removed and replaced with 0.5 ml of MEM per animal (3 ml) and triturated a third time.

The final cell suspension was plated on poly-L-lysine coated dishes and placed at 37°C with 5% CO_2 _in an incubator for 60 minutes before addition of 2 ml of MEM medium. The cultures were incubated for 2–3 days before the MEM medium was removed and replaced with neurobasal medium contain 50 μM glutamate. The cultures were incubated for a further 2 – 3 days before being used in experiments and all the cultures used in this study were in culture for 5 to 10 days.

### Electrophysiology

The cultures were removed from the neurobasal culture medium and washed twice with 1 ml extracellular NaCl-based extracellular solution. All experiments were conducted at room temperature ~21°C. The whole cell recording technique [15] was used to measure changes in the electrophysiological properties of neurones in response to application of test compounds. Membrane potential, input resistance and holding current were measured via a low resistance (4 – 10 MΩ) borosilicate patch pipette pulled on Kopf model 730, needle puller and back-filled with KCl-based patch pipette filling solution. An Axoclamp-2A switching voltage clamp amplifier operated at sampling rate 15–20 kHz was used. Poly-APS and synthetic compounds were applied to neurones by low-pressure ejection from a blunted borosilicate patch pipette positioned approximately 100 μm away from the target cell being studied. After drug application had stopped, compounds were allowed to diffuse in the surrounding bath medium to a negligible concentration as a means of drug removal. A maximum of two sequential experiments were conducted using the same culture dish, cells were washed twice between experiments and target cells selected from two distant regions of the dishes.

To standardise some measurements of electrotonic potentials (for input resistance) and action potentials, neurones were held at -70 mV and depolarising and hyperpolarising current step commands (100 – 300 ms) applied in the absence and presence of poly-APS or a synthetic compound. Single electrode voltage clamp was used to measure currents activated by poly-APS and the synthetic compounds. Currents were activated from a holding potential of -70 mV and voltage step commands (100 ms) were applied to generate current-voltage relationships between -140 and -70 mV. All data were recorded and stored on digital audio tape (DTR 1200, Biologic). Data were retrieved and analysed using CED Vclamp software, where appropriate example traces were recorded using a chart recorder (Gould pen recorder, 2200S). Statistical analysis was conducted using Prism software, 2 way ANOVA, Bonferroni post test and Student's two- tailed, paired t-tests were used. All data are given as means ± standard error of the mean with associated *p *values where relevant.

### Calcium Imaging

Hippocampal cultures were washed with HBS at room temperature and loaded with the cell-permeable fluorescent calcium indicator fura-2-AM (10 μM from a 1 mM stock solution in dimethylformamide, Molecular Probes, OR, USA) [16] in 1 ml HBS, for 1 hour in the dark. The intracellular Ca^2+ ^imaging experiments where conducted as previously described [6,7,9]. Cultures were perfused with HBS at a rate of 1 – 2 ml/min, using a gravity perfusion system. Images were collected at 1 frame every 3 seconds using Oracal software. All experiments were replicated at least 3 times using dishes from 3 different cultures. The statistical significance of the data were tested using Kruskal-Wallis test and Dunns multiple comparison tests, *p *values are given where relevant.

### Membrane Fluidity Measurements

Membrane fluidity was quantified by steady-state fluorescence anisotropy using the probe 1,6-diphenyl-1,3,5-hexatriene (DPH) [17-19]. The incorporation of this probe into membranes is accompanied by a strong enhancement of the fluorescent signal. Intercalation of DPH between lipid acyl chains also enables the measurement of alterations of membrane order along the lipid acyl chain axis at deeper portions of the bilayer hydrophobic region [18].

Hippocampal cell cultures were loaded with 100 μM DPH (Molecular Probes) in MEM for 1 hour at room temperature, in the dark. Cultures were then washed and perfused with HEPES buffered solution (composition in mM: NaCl 130; KCl 5.4; CaCl_2 _1.8; MgCl_2 _0.1; HEPES 10; glucose 25), at a rate of 1–2 ml/min, using a gravity perfusion system.

A suitable field of cells was identified and excitation and emission wavelengths of 360 nm and 450 nm respectively were used to image DPH fluorescence. Approximately 20 images were captured per minute. Poly-APS were applied at different concentrations for two minute periods. Run-down in fluorescence intensity occurred rather rapidly due to the instability of DPH within the membrane this was accounted for during analysis by subtracting the gradient of run-down for each region of interest (taken as a change in fluorescence intensity between two points prior to any drug application, see Figure 8) from any change that occurred in membrane fluidity during application of poly-APS. DMSO was used as a standard solvation reagent to test phase stability of cell membranes [12]. All experiments were replicated at least 3 times using dishes from 3 separate cultures. Statistical significance was conducted using Kruskal-Wallis test and Dunns multiple comparison tests, *p *values are given where relevant.

### Preparation of liposomes for surface plasmon resonance

Cholesterol/sphingomyelin large unilamellar vesicles (LUVs) in a 1/1 molar ratio were formed by removing the organic solvents from the lipid solution in a rounded flask with rotary evaporation and final vacuum drying. Lipid film, at a final concentration range of 1 mg/ml, was swelled in vesicle buffer (140 mM NaCl, 20 mM TRIS, 1 mM EDTA, pH 8.0), and vortexed vigorously to obtain multilamellar vesicles. To obtain LUVs of a defined size, the suspension of multilamellar vesicles was subjected to 8 freeze-thawing cycles, and extruded through 0.1 μm polycarbonate filters (Avestin, Ottawa, Canada) at 50°C in the thermostatically controlled water bath.

Lipid concentration was determined colorimetrically with Waco Free Cholesterol C and Wako test Phospholipids B (990–54009) kits (Waco Chemicals GmbH, Germany).

### Surface plasmon resonance (SPR) measurements

Binding of poly-APS to immobilized cholesterol/sphingomyelin (1/1) LUVs was determined using a Biacore × SPR apparatus and an L1 Sensor Chip (Biacore AB, Uppsala, Sweden), that retains intact liposomes on its lipophilic surface [20]. Using this technique, we were able to directly monitor the interaction between poly-APS and lipid bilayers. All ligands and washing chemicals were prepared in the degassed vesicle buffer, which was previously filtered through a 0.22 μm filter, and which was also used as a running buffer. 10 μL of LUVs (0.5 mM) were deposited on the chip surface at a flow rate of 1 μL/min to reach a response of 11000–11500 resonance units (RU). Loosely adsorbed lipid materials were washed out successively with 100 μL of 100 mM NaOH (30 μL/min) and with running buffer. 100 μL of poly-APS (0.005 or 0.5 μg/ml in the running buffer) were subsequently injected over immobilized LUVs at a flow rate of 30 μL/min, followed by the continuous flow of the running buffer to obtain dissociation kinetics. Sensorgrams were recorded at 25°C. At the end of experiments, the chip surface was purged with 100 μL of isopropanol: 100 mM NaOH (2:3, v/v), and fresh LUVs were applied following the above procedure.

Controls were run by applying poly-APS alone to the chip surface, and bulk controls were run on immobilized vesicles with vesicle buffer alone. Sensorgrams were processed and evaluated using the BIAevaluation Version 3.2 software (Biacore AB, Uppsala, Sweden). No fitting of the experimental curves was performed, due to the complex association/dissociation kinetics.

## Abbreviations

DPH 1,6-diphenyl-1,3,5-hexatriene

DRG dorsal root ganglion

LUVs large unilamellar vesicles

Poly-APS polymeric alkylpyridinium salts

siRNA small interfering RNA

SPR Surface plasmon resonance

## Authors' contributions

IM & GG carried out the synthetic chemistry to produce the test compounds (1, 2, 3 & 4). KS, TT & KR isolated the poly-APS and carried the surface plasmon resonance experiments. KH, GR & BP conducted the membrane fluidity study. DJK, KCD, KH & RHS carried out the calcium imaging experiments. DJK, KCD & RHS conducted the electrophysiology. All authors contributed to the projects design and write up.
